# Estimation of the Allergenic Potential of Urban Trees and Urban Parks: Towards the Healthy Design of Urban Green Spaces of the Future

**DOI:** 10.3390/ijerph16081357

**Published:** 2019-04-15

**Authors:** Paloma Cariñanos, Filipa Grilo, Pedro Pinho, Manuel Casares-Porcel, Cristina Branquinho, Nezha Acil, Maria Beatrice Andreucci, Andreia Anjos, Pietro Massimiliano Bianco, Silvia Brini, Pedro Calaza-Martínez, Enrico Calvo, Elisa Carrari, José Castro, Anna Chiesura, Otilia Correia, Artur Gonçalves, Paula Gonçalves, Teresa Mexia, Marzia Mirabile, Elena Paoletti, Margarida Santos-Reis, Paolo Semenzato, Ursa Vilhar

**Affiliations:** 1 Department of Botany, University of Granada, 18071 Granada, Spain; mcasares@ugr.es; 2 Andalusian Institute for Earth System Research (IISTA-CEAMA), University of Granada, 18071 Granada, Spain; 3 Centre for Ecology, Evolution and Environmental Changes (cE3c), Faculdade de Ciencias da Universidade de Lisboa, 1749-016 Lisbon, Portugal; afgrilo@fc.ul.pt (F.G.); paplopes@fc.ul.pt (P.P.); cmbranquinho@fc.ul.pt (C.B.); asanjos@fc.ul.pt (A.A.); odgato@fc.ul.pt (O.C.); pigoncalves@fc.ul.pt (P.G.); teresa.mexia@gmail.com (T.M.); mmreis@fc.ul.pt (M.S.-R.); 4 School of Geography, Earth and Environmental Science and Birmingham Institute of Forest Research, University of Birmingham, Birmingham B15 2TT, UK; nezha.acil@gmail.com; 5 Faculty of Architecture, Sapienza Universitá di Roma, 00185 Rome, Italy; mbeatrice.andreucci@uniroma1.it; 6 Institute for Environmental Protection and Research (ISPRA), 00144 Rome, Italy; pietro.bianco@isprambiente.it (P.M.B.); silvia.brini@isprambiente.it (S.B.); anna.chiesura@isprambiente.it (A.C.); marzia.mirabile@isprambiente.it (M.M.); 7 Spanish Association for Public Parks and Gardens, 28223 Pozuelo de Alarcón, Madrid, Spain; calaza@iies.es; 8 Regional Agency for the Service of Agricultural and Forest (ERSAF), 2014 Milano, Italy; Enrico.calvo@ersaf.lombardia.it; 9 CNR Sesto Fiorentino, 50019 Florence, Italy; eli.carrari@gmail.com (E.C.); elena.paoletti@cnr.it (E.P.); 10 Centro de Investigação da Montanha (CIMO), Instituto Politécnico de Bragança, 5300-253 Bragança, Portugal; jose.ferreiradecastro@fao.org (J.C.); ajg@ipb.pt (A.G.); 11 Department of Land and Agro-Forestry Systems, University of Padova, 35020 Legnano, Italy; paolo.semenzato@unipd.it; 12 Slovenian Forestry Institute, 1000 Ljubljana, Slovenia; ursa.vilhar@gozdis.si

**Keywords:** index of urban green zone allergenicity, cost of greening, impact on health, ecosystem disservices, allergenic trees, value of potential allergenicity (VPA)

## Abstract

The impact of allergens emitted by urban green spaces on health is one of the main disservices of ecosystems. The objective of this work is to establish the potential allergenic value of some tree species in urban environments, so that the allergenicity of green spaces can be estimated through application of the Index of Urban Green Zones Allergenicity (I_UGZA_). Multiple types of green spaces in Mediterranean cities were selected for the estimation of I_UGZ_. The results show that some of the ornamental species native to the Mediterranean are among the main causative agents of allergy in the population; in particular, Oleaceae, Cupressaceae, Fagaceae, and *Platanus hispanica*. Variables of the strongest impact on I_UGZA_ were the bioclimatic characteristics of the territory and design aspects, such as the density of trees and the number of species. We concluded that the methodology to assess the allergenicity associated with urban trees and urban areas presented in this work opens new perspectives in the design and planning of urban green spaces, pointing out the need to consider the potential allergenicity of a species when selecting plant material to be used in cities. Only then can urban green areas be inclusive spaces, in terms of public health.

## 1. Introduction

Urban green spaces (UGS) are of strategic importance to the quality of life of urban dwellers [1,2]. They provide a series of ecosystem services (ES) that have direct and indirect effects on public health [3]. The direct effects include all those processes that mitigate environmental degradation: air purification [4,5,6], carbon sequestration [7], climate regulation [8], and water regulation and purification [9,10]; and those that directly prevent diseases—improved psychological well-being [11] and reduced stress [12]. The indirect effects are related to the possibility offered by UGS to carry out physical activities and sports [13], i.e., reduced obesity and cardiovascular symptoms [13,14], leisure and recreation [15,16], socialization, and contact with nature [17], which result in a feeling of well-being that contributes to improved health and quality of life [18].

When establishing the net balance of benefits that UGS provide, we must, however, consider the negative factors emanating from natural functions of ecosystems or their anthropogenic manipulation [19]. These negative effects, defined by some authors as ecosystems disservices (ED) [20,21], generate important environmental and socioeconomic costs, and sometimes have a great impact on health [22]. The adverse reactions caused by the emission of allergenic pollen during flowering of the plant species that form the UGS are one of the main EDs, with a high effect on citizens’ welfare [23]. Approximately 30% of the world population is affected by reactions caused by allergenic pollen in the atmosphere [24]. In Europe, more than 150 million citizens suffer from chronic allergic diseases, with an estimated cost between 55-151 billion euros/year [25] to the National Health Services. This issue constitutes one of the main public health burdens today, and is expected to increase exponentially in the coming years as a result of the effects of climate change and growing urbanization, industrialization, and pollution [26].

In the urban context, UGS have been identified as the main source of allergen emission [27] and are among the factors triggering allergy symptoms in city dwellers [28,29]. Climatic, ecological and geographical characteristics play an important role in the allergens prevalent in each territory [30], by affecting the zonal distribution of vegetation [31], both in natural and urban environments. This has allowed establishing of the geographic range of distribution of the major allergens: some of them are of world-wide distribution, such as grasses, as they are favored for their great adaptability and participation in many varieties of lawns and numerous urban green elements [32]. Closer relationships between allergens and climatic conditions occur when the emitting species are those best represented in the considered bioclimate, such as olive tree (*Olea europaea*) in the Mediterranean region [33], *Cryptomeria japonica* in Japan [34], birch (*Betula* spp.) in the northern Europe [35], maples (*Acer* spp.) and willows (*Salix* spp.) in Canada [36], or several species of Myrtaceae in Australia and New Zealand [37].

A major priority is thus to provide citizens and administrators with tools and mechanisms that allow for the adoption of preventive measures and reduction of the negative impacts that the presence of allergenic pollen may have on the sensitive population. The sampling of atmospheric allergens and the subsequent dissemination of information constitute one of the main strategies to raise awareness [38]. Several European cities have an aerobiological sampling unit (https://www.zaum-online.de/pollen/pollen-monitoring-map-of-the-world.html), but for the most part, the levels recorded are too general, over a wide area of coverage [39,40], and do not represent situations closest to the population (i.e., breathable air at human height). There are also attempts being made to assess the risk that the presence of allergen-emitting plants may have on the population, and to identify and categorize the allergenic level of different plant species [41,42,43]. Some studies have reviewed the causes of the increasing allergenicity of UGS, and pointed out the low biodiversity of species used, the introduction of exotic species, the discrimination of female specimens or the cross-reactions that are established between phylogenetically related species as main causes [44]. However, if there is a cause that should be highlighted, it is the non-consideration of the criterion of allergenicity when selecting urban plant material. This lack of planning in the design of green areas results in severe health risks at certain periods of the year.

This work aims to establish the potential allergenic value of some of the most common tree species in urban environments, so that the allergenic risk generated in the UGS can be easily estimated. Different typology of green spaces, encompassing a climate gradient, was also considered in order to analyze the design and infrastructure characteristics and parameters that may impact the quality of UGS in terms of health benefits. The results will prevent allergy sufferers from situations that may pose a risk to their health.

## 2. Materials and Methods

### 2.1. Selection of Urban Parks and Inventory of Vegetation

**Figure 1:**
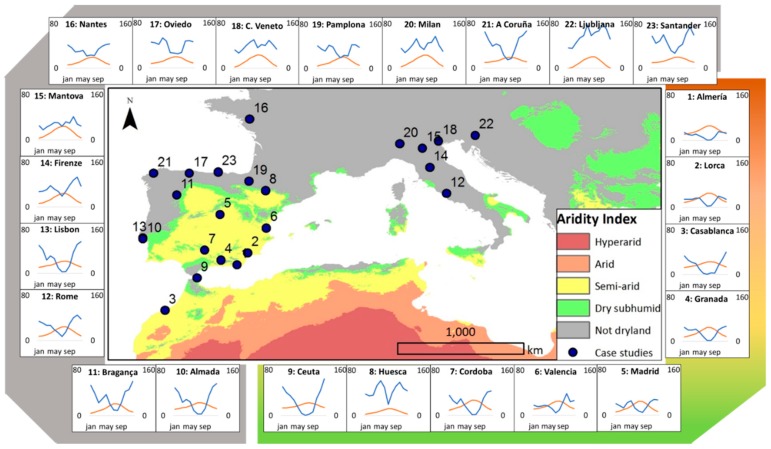
Map and climatic characteristics of the cities participating in this study. Left axis in climatic graphs represents monthly air temperature in °C; the right axis represents monthly precipitation in mm, and the horizontal axis represents the time in months. General characteristics of the parks (coordinates, surface area, turf area, number of trees, number of species and density of trees) are listed in SI Table S1.

A selection of urban parks was carried out, across a range of characteristics, in terms of typology, size, design and style (both historical and modern) [45,46], in 23 cities from six Mediterranean countries: France, Italy, Morocco, Portugal, Spain, and Slovenia. In order to analyze the existing diversity in the same country, several cities in Italy, Portugal, and Spain were considered. The type of park and its location within the city may be related to the type and frequency of activities that are carried out in it [13]. Therefore, location and general characteristics of these urban parks are detailed in Figure 1 and Table S1.

An inventory of the existing species was carried out, either by details from the Park and Gardens Services of the municipalities, as provided by local institutions, or by direct in situ field surveys and identification, by staff collaborators. Reference literature and monographic floras were used to determine the species [47,48,49,50]. The vegetation inventory included taxonomic determination at the species level (cultivar or variety if possible), and the reproductive character, including hermaphrodite, monoecious, or dioecious species. In the latter case, the number of specimens of each sex was considered, given the allergenic implications when only male specimens are planted. The deciduous or evergreen character was also considered, because it may affect the dispersion of pollen grains. In addition, the geographic origin of the tree species was established, due to the interest that this information can have when determining the causative agents of major allergies. The hardiness zone category, which provides information about the ability of different species to withstand minimum temperatures of the zone, was also determined [51]. The presence of cover grass was finally taken into account, as this information affects the final value of the allergenic risk.

### 2.2. Allergenic Risk Assessment

In order to estimate the potential allergenic risk of urban green areas, the Index of Urban Green Zone Allergenicity (I_UGZA_), proposed by [52] was applied, which is related to the time of flowering and varies between 0 and 1. The index combines a series of biological and biometric parameters of the different species of trees, according to the formula:

$$I_{UGZA}=\frac{1}{maxVPAxS_{T}}\overset{k}{\underset{i=1}{\sum }}VPA\times S_{i}\times Hi$$

where: VPA = Value of Potential Allergenicity of each species; *S_T_* = Surface of the urban park; *k* = number of species in the park; *S_i_* = Area occupied by each species in the park; *H_i_* = maximum height that the tree can reach at maturity [53].

VPA results from the combination of three parameters intrinsic to the species: pollination strategy (ps), duration of the pollination period (dpp), and intrinsic allergenic capacity of the pollen grains (ap) [52,53]. To collect the information regarding the parameters of each species, we consulted numerous documentary sources from different disciplines such as taxonomy, botany, forestry, or allergology. With this information, a database of parameters for the calculation of the I_UGZA_ was created (SafeCreative code 1803156149680, IPR-684).

The VPA of each species is then combined with the average allometric parameters, i.e., the average volume of tree canopy emitting allergens that each tree species will have upon reaching the reproductive maturity. This volume of allergen emission is calculated from *S_i_* and *H_i_*. By extending the result to *S_T_*, it is possible to know the contribution of each species to the total allergenicity of the UGS. It can also identify the species that have a greater contribution in terms of surface area or abundance of existing trees. Finally, for each park, the percentage of grass covered area was calculated, since this data may have an impact on the final allergenicity value.

The analysis of the different situations of allergenicity that can be generated in a park defined a value of I_UGZA_ of 0.3 as a threshold to establish the risk that the presence of allergenic species in these parks can represent for allergic people [54]. This threshold of 0.3 can be recorded when there are monospecific formations of allergenic species in a park, or there are planted species of moderate allergenicity among which cross-reactions can be established, or when the percentage of allergenic species in the park is higher than that of the non-allergenic species. Based on this value, the parks were classified as low (<0.2), moderate (0.2–0.3), or high allergenicity (>0.3).

### 2.3. Data Analysis

In order to identify the factors with the strongest impact on the I_UGZA_ of the different parks, a set of environmental variables was analysed. For bioclimatic variables, we used air temperature and rainfall as they are biologically meaningful [55]. The values (average 1970–2000) were retrieved from Worldclim.org for city centres, being coded as follows: annual mean temperature (BIO1), mean diurnal range (BIO2), isothermality (BIO3), temperature seasonality (BIO4), maximum temperature of the warmest month (BIO5), minimum temperature of the coldest month (BIO6), annual temperature range (BIO7), mean temperature of the wettest quarter (BIO8), mean temperature of the driest quarter (BIO9), mean temperature of the warmest quarter (BIO10), mean temperature of the coldest quarter (BIO11), annual precipitation (BIO12), precipitation of the wettest month (BIO13), precipitation of the driest month (BIO14), seasonality precipitation (BIO15), precipitation of the wettest quarter (BIO16), precipitation of the driest quarter (BIO17), precipitation of the warmest quarter (BIO18), and precipitation of the coldest quarter (BIO19) (Figure 1).

Tree density and Shannon diversity index [54] were also calculated for each park. Firstly, an exploratory analysis was performed in order to understand which variables significantly contributes to the I_UGZA_ index. To do so, individual variables were tested with Spearman correlations and the most significant results were then submitted to a generalized linear model (GLM) with identity link (and normal distribution). All possible combinations of those significant variables were tested. Looking for the best possible model and using the parsimony principle, the model with the highest variance and with a significant contribution of all predictors was selected for further interpretation. All statistical analyses were implemented in Statistica^TM^ software (Tibco Software Inc., Palo Alto, CA, USA). Finally, the main taxa contributing to high I_UGZA_ values were identified.

## 3. Results

The parks included “Large Urban Parks”, “Urban Forests”, “Historical Gardens/Parks”, “Community Parks”, “Pocket Parks”, “Botanical Gardens”, “Boulevards”, and “Promenades” (Table S1). The range of sizes included parks larger than 100 ha (El Retiro, Madrid), between 10–50 ha (e.g., Bosco dei Cento Passi, Milan, Parco di Arlecchino, Mantua, Parco Talenti and Parco del Colle Oppio, Rome, Parque da Paz, Almada, or Parque Las Llamas, Santander), and smaller than 10 ha (Miklosicev in Ljubljana, San Amaro Park in Ceuta or Castle Park in Bragança).

The surface covered by grass ranged from 0% in the most arid site (Almeria), to more than 95% in some of the 12 parks in Rome. In absolute terms, the most extensive grass surfaces were found in Talenti Park (110,000 m^2^), Parque da Paz, Almada (273,772m^2^ between irrigated and non-irrigated meadows), and El Retiro Park (with more than 300,000 m^2^).

The total number of taxa was 355 in terms of species, sub-species, and varieties, from 83 botanical families, for a total of more than 110,000 trees (Table 1). The largest number of species was registered in the Jardin des Plantes, with 160 species. The Jardim Guerra Junqueiro and El Retiro Park also exceeded 100 species (Table S1).

In relation to sexual attributes, 62.2% of the species had both sexes on the same individual (hermaphrodite, monoecious), while 67 species, i.e., 18.8%, had separated sexes in different individuals (dioecious) (Table 2). Regarding the strategy of pollination, 46.7% of the species were insect-pollinated. By contrast, 42.3% of the plants used the wind as vector of pollination. The deciduous attribute was present in 65.0% of the species, while 33.1% were evergreen.

**Figure 2:**
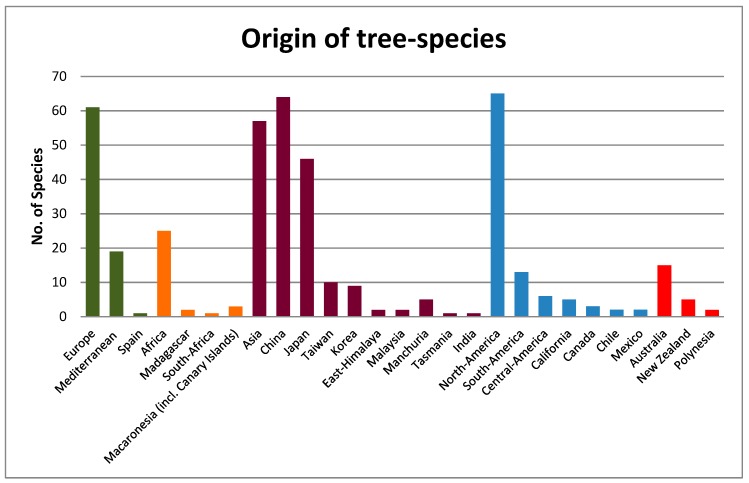
Geographical origin of the tree species found in the parks.

**Figure 3:**
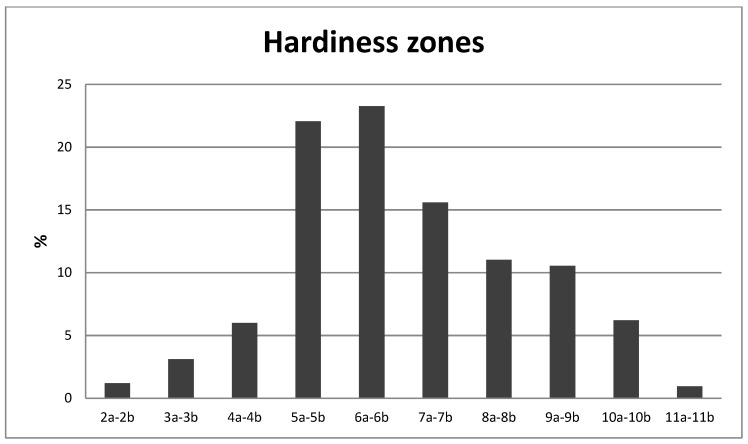
Distribution of tree species found in the parks according to their hardiness zone. 2a–2b: −45.6 to −40.0 °C; 3a–3b: −40.0 to −34.4 °C; 4a–4b: −34.4 to −28.9 °C; 5a–5b: −28.9 to −23.3 °C; 6a–6b: −23.3 to −17.8 °C; 7a–7b: −17.8 to −12.2 °C; 8a–8b: −12.2 to −6.7 °C; 9a–9b: −6.7 to −1.1 °C; 10a–10b: −1.1 to +4.4 °C; 11a–11b: +4.4 to +10.0 °C.

In relation to the origin of the species, 18.3% of them were native to North America; 18.0% were of Chinese origin, and 17.0% were native to Europe, of which 5.4% were of Mediterranean origin (Figure 2). As for the hardiness zone categories (Figure 3), 60.9% were able to tolerate minimum temperatures ranging from −28.9 °C to −12.2 °C (categories 5, 6 and 7), 28.8% were included in the range of categories 8 to 11 (from −12.2 °C to + 4.4 °C), while a group of 43 plants (10.3%) were able to tolerate minimum temperatures below −35 °C (categories 2 to 4).

**Figure 4:**
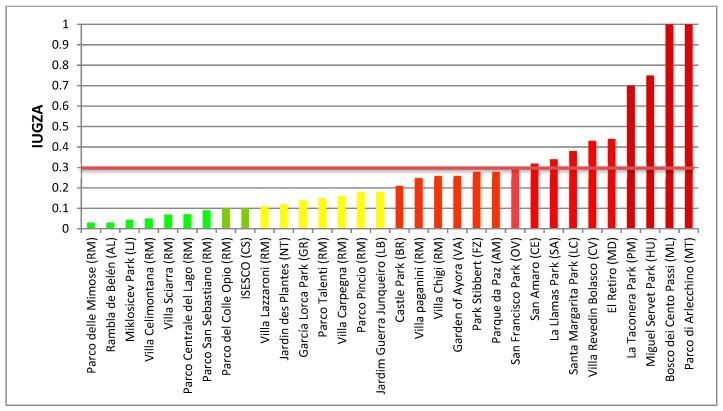
Index of Allergenicity of Urban Green Areas (I_UGZA_) of the selected parks. RM: Rome; AL: Almeria; LI: Ljubljana: CS: Casablanca; NT: Nantes; GR: Granada; LB: Lisbon; BR: Bragança; VA: Valencia; FZ: Florence; AM: Almada; OV: Oviedo; CE: Ceuta; SA: Santander; LC: La Coruña; MD: Madrid; PM: Pamplona; HU: Huesca; ML: Milan; MT: Mantua.

In the list of the 20 most frequent species (Table 3), most species were native to the European continent with the exception of *Acer negundo*, *Magnolia*, and *Robinia pseudoacacia*, which were from North American, and *Ligustrum lucidum*, originally from China. As for the attributes, the majority were monoecious (7) or dioecious (7), wind-pollinated (13), and deciduous (12). The application of the I_UGZA_ (Figure 4) revealed that 10 parks exceeded the threshold value of 0.3, resulting in severe potential health risks during specific periods of the year. Two of the parks, Parco di Arlecchino and Bosco dei Cento Passi, registered the maximum possible value of I_UGZA_, i.e., 1, so the risk of suffering allergic symptoms is maximum.

Regarding the variables that have the greatest impact on the value of the I_UGZA_, certain structural characteristics of the parks such as the number of trees and of species, the Shannon’s index and the number of trees/ha^−1^ and also precipitation of May and July, precipitation of the warmest quarter (BIO 18), annual mean temperatures (BIO 1), and mean temperature of the driest quarter (BIO 9) significantly affected I_UGZA_ (Table 4). Density of trees was one of the parameters with the highest positive correlation with the value of I_UGZA_ (*r* = 0.70; *p* < 0.01) (Table 5), and accounted for most of the variance as a predictor in the tested GL model (adjR^2^ = 076).

## 4. Discussion

In this work, 34 parks located in 23 Mediterranean cities were considered, so that the spaces in which Mediterranean citizens perform outdoor activities were well represented [45,46]. The study showed important information that can affect the allergenic impact of Mediterranean UGS on citizens’ health. First, the type, size and location within the city of the park helped to predict I_UGZA_ and thus can be used for programming the frequency and duration of the visits. We considered some small parks that are usually located in city centers and historical districts, densely built districts, or in the vicinity of administrative or monumental buildings. In these small parks, there is a large presence of citizens who perform daily activities: sport routines, socialization between similar age groups, pet walking or relaxation [55]; thus, during the period of flowering, the contact and interactions with the allergens and other atmospheric particulate matter are frequent [56]. This is the case of parks in the city of Rome or El Retiro in Madrid, in which the maximum number of local visitors and tourists at the beginning of spring coincides with the period of flowering of *Platanus* and the species of Oleaceae, Fagaceae and Pinaceae [57,58]; people should, thus, be warned so they could take precautionary measures.

The inventory of vegetation revealed the extraordinary rich and varied native flora of this climatic region [59], which also allows for the growth of other taxa from other geographical origins and phytoclima [60,61]. A good index of the diversity of the parks was indicated by their high number of taxa (355) with a total of more than 110,000 trees. This figure contrasts with those obtained for other areas of Europe, since a study on the diversity and distribution of trees in 10 major Nordic cities showed a markedly lower diversity (133 different tree species for the city with the highest diversity), with a total number of trees exceeding 190,000, including street and park trees [62]. The largest number of species was registered in the Jardin des Plantes (Nantes), given its arboretum and botanical garden character, although other parks also exceeded 100 species, making these urban green zones true biodiversity hotspots in the urban environment [61,63].

This great diversity was also reflected in aspects such as sexual attributes and pollination strategies. Regarding the latter, most of the entomophilous species had bees as a main pollinator agent [64]. This confirms the important role of urban parks in the provision of ES, not only because of the diversity of bee species that participate in pollination, but because this regulating service is essential to maintaining ecosystem processes [65]. By contrast, the anemophilous strategy, in which the wind is the driver of pollination, is the cause of one of the main disservices associated with urban vegetation [22]. This process of anemophilia is even more intense when considering the coniferous species, as all of them are primary anemophilic, and the deciduous anemophilic angiosperms [66], which developed this strategy in a later evolutionary phase and adjusted the functional process from anthesis to the moment immediately before the new leaves unfold, so that the emission of pollen is made from the anthers without any obstacle.

The 20 most frequent species included species native to the European continent, some of Mediterranean origin, which led us to consider them as major allergens in the region. This group included some of the main causative agents of pollen allergy in the Mediterranean area, such as *Cupressus* [67], and *Platanus x hispanica* [68], both with a very high VPA. Other species characteristic of the Mediterranean, such as *Pinus halepensis*, *Pinus pinea*, and *Quercus ilex*, were also included in the list, although with a lower degree of allergenicity, all of them largely distributed in some cities such as Rome [69]. These results are in line with those obtained in a previous study on the allergenicity of the ornamental flora carried out in two Mediterranean cities [70], but contrast sharply with those from other European regions, where there was a clear prevalence of species of the genera *Tilia*, *Acer*, *Aesculus*, and *Fraxinus* in Central and Continental Europe [71], and *Betula*, *Sorbus*, *Carpinus*, and *Fagus* in northern Europe [62,72].

Plane tree (*Platanus x hispanica*) is one of the most notable species due to its extensive presence in European cities and the rest of the world [71]. In our study, plane tree was recorded in 95% of the inventories, with an unequal presence, and therefore an unequal contribution to the final value of I_UGZA_. Thus, the overabundance of individuals in some of the Spanish parks (more than 400 in Huesca and Pamplona, and almost 1000 in Madrid) had a very high contribution to the value of I_UGZA_. Although this pollen type has low dispersion capacity, estimated to be just 400 m from the source [73], the tree’s deciduous character favors the dispersion before the new leaves begin to develop [74]. By contrast, this tree species has a short flowering period, which limits the time of emission to just a few weeks [68]. *Cupressus sempervirens* (Tuscany cypress) is another frequent species in Mediterranean urban parks. Its very high value of allergenic potential can be applicable to the rest of the species of the family Cupressaceae [67]. All of them share reproductive attributes such as anemophilic character, high pollen production [75], extensive flowering period [76], and very high allergenic pollen grains, thus being one of the allergenic-type typical of the Mediterranean region [77]. Where their presence is abundant, authorities should warn the population during their pollination period, which usually occurs during the winter months.

Several species of Oleaceae family must be pointed out. The different species of *Fraxinus* may have different reproductive attributes. Two of the most frequent species, *F. excelsior* and *F. angustifolia*, are dioecious and wind-pollinated, so that a greater presence of male individuals can influence not only VPA but also the amount of pollen emitted [78]. In contrast, Privet (*Ligustrum* sp.) is an insect-pollinated Oleaceae species, but little amounts of pollen emitted may be sufficient to cause allergy reactions if a person stays in its vicinity [79]. Finally, it is necessary to stress that the presence of *Olea europea* in urban parks is increasingly frequent. In our study, several olive trees grew in parks in Italy, Spain and Portugal, some with centenary specimens. In addition to its pollen grains being the first cause of pollen allergy in the Mediterranean region [80], we must consider the cross-reactions that can be established between the different species of the family due to the presence of shared allergens [81].

Another family with Mediterranean species is Pinaceae. Although the allergenicity of its pollen grains is low [82], it is pertinent to recall other sensitivity reactions that can be generated by the presence of caterpillars [83]. Orange trees (*Citrus aurantium*) deserve particular attention, as they emit sufficient pollen levels to generate a symptomatic response in the population, due to their relatively high frequency in parks and streets of Mediterranean cities [68]. *Populus alba* is one of the few species of *Populus* of European origin. Given the existence of exclusively male-sex clones, allergenicity is linked to a greater or lesser presence of male individuals [84]. This list also includes some species that tolerate minimum temperatures below −20 °C (hardiness zones 4a–5b). *Acer negundo* is the only wind-pollinated species of the genus, which thus increases its allergenicity to high [85]. *Taxus baccata* has a phylogenetic link with the Cupressaceae family, with which it shares allergens and allergenicity [86]. Linden (*Tilia* spp.) is widely used in walk-alignments, so that its moderate allergenicity can be increased when forming dense groups [87].

A relevant aspect of this work was to establish the allergenic risk assessment of 34 parks and the factors that pose the greatest hazards. A value of I_UGZA_ index of 0.3 had been established in a previous work as the threshold above which the presence of allergenic plants is high enough to cause discomfort and symptoms to an allergic population [53]. In our study, 10 parks exceeded this threshold, and two parks (Parco di Arlecchino in Mantua and Bosco dei Cento Passi in Milan), registered the maximum value of I_UGZA_. Reviewing the characteristics of these last parks, most of them showed a density of trees higher than 150 trees/ha, with peaks of 562.6 trees/ha and 771 trees/ha. This relationship between I_UGZA_ and density of trees was already evident in a previous work [53], but in this work, it has been reinforced by the characteristics of the main species of these parks: *Carpinus betulus* and *Quercus robur*. The allometric parameters of both species in terms of the area they occupy in relation to the surface of the park (S_i_/S_T_ ratio) and crown height generate a volume of tree canopy emitting allergens that, together with the high density of existing trees, take the value of I_UGZA_ to its maximum. The species richness and the Shannon Index were also correlated with I_UGZA_. Surprisingly, the correlations were positive. This relationship is clear when there is variety and equity among the species in the parks [54], since it would have a balanced diversity among species with different flowering and allergenicity attributes. However, an imbalance in this ratio could take the index to maximum values (Bosco dei Cento Passi: I_UGZA_: 1, Species richness: 15, Shannon’s Index: 3.16), or minimum, as in the case of the Parco Centrale del Lago, in Rome, which with its 935 trees of 50 different species, Shannon’s Index: 3.3. register some of the lowest I_UGZA_ values, 0.07.

The latitudinal gradient can also be considered an important parameter, since it determines the prevailing environmental variables in each zone. In our study, we analysed parks on both shores of the Mediterranean, from 33° N in Casablanca to 47° N in Nantes, resulting in a large span of local climatic conditions [88]. The Spearman Correlation revealed that temperature and precipitation significantly affected the I_UGZA_ index. Both parameters are closely related to the flowering of plants [89], and pollen emissions [90], but here the sign of the correlations was opposite i.e., negative. In the Mediterranean area, the occurrence of rainfall during the warmest period (BIO18) and even during the flowering period (BIO15), prevents water stress and favors a more intense flowering [91]. As for mean temperatures, its correlation is negative. The inclusion of the annual mean temperature (BIO 1) in the model as one of the most significant variables suggests that the value of the I_UGZA_ is higher in green spaces of colder cities. This aspect was reflected in the floristic composition: parks located in the northern regions of Portugal, Spain, France, and Slovenia had among their most contributory species to the index some taxa of temperate climates such as *Carpinus betulus*, *Corylus* spp., *Taxus baccata*, *Fagus sylvatica*, *Pterocarya* sp., and deciduous *Quercus*. All species of these genera are included in the hardiness zone categories (5a to 6b), suggesting that they can tolerate minimum temperatures below −20 °C. In addition, we postulate that a landscape design forest-type, with high density of tree ha^−1^, and a greater presence of monospecific groupings increased the magnitude of pollen emission, and contributed to the remarkable increase in the I_UGZA_ index. In the parks of Rome, Casablanca, and the south of Spain, species of Oleaceae, Cupressaceae, *Pinus*, and evergreen *Quercus* were abundant, which are more tolerant to thermophilic conditions and aridity, and are included in categories 8a to 10b of hardiness zones. This distinctive floristic composition affected I_UGZA_, since it is also true that, in general, the highest values of species richness and the Shannon Index were recorded in the parks of the most temperate zones.

Another element with an impact on the final value of I_UGZA_ is the total area covered by grasses. The majority of the lawns covering the parks can be considered as conventional, that is, requiring periodic management, including frequent cuts and regular irrigation [92]. There is evidence of allergic symptomatology in lawn cutters [93]. Drought-tolerant, summer growth and high allergenicity species, such as Bermuda grass (*Cynodon dactylon*) and kikuyu grass (*Pennisetum clandestinum*) were often abundant [94,95]. The more and more frequent presence in Mediterranean parks of natural grasslands or grass of low hydric requirements [96] is favoring in turn the installation in green areas of grasses of great colonizing and allergenic capacity [97]. In the parks of this study, the contribution to the I_UGZA_ index value of the turf covered area was particularly significant in some parks in Rome, Stibbbert park in Florence, Portuguese Parque da Paz and El Retiro Park in Madrid.

As a final remark, we would like to emphasize that the results of this study should not be interpreted from a negative perspective, but as information to take into account when making the net balance of ES provided by the elements of urban forest [98]. Many of the cities that participated in this study have an important historical and cultural past, which has survived to this day in the form of historic parks and gardens [99]; while other cities have experienced important urban transformations, in which the greening process has changed the urban landscape to a great extent [100]. Whatever the case, all cities must face the challenge of climate change, mitigate its impact, and reinforce its resilience. In this context, green infrastructure in general, and urban parks in particular will play a fundamental role as providers of ES, and benefits and well-being in the population [2,4]. The growth expectations of diseases related to environmental degradation, including respiratory diseases [26], highlight the need to implement plans aimed at reducing the risk of allergenicity and improving public health. The information resulting from this study opens a frontier of knowledge to the fact that green spaces are inclusive spaces in term of health without limitations in the presence of specific qualities provided to urban built environments.

## 5. Conclusions

This work presented a methodology to assess the allergenicity associated with urban trees and urban areas of different cities in the Mediterranean region, although the high number of species analyzed allows its application to other bioclimatic regions. The results indicated that the species that present a series of attributes, such as monoecia or dioecia, wind-pollination, deciduous and extensive periods of flowering, are the ones with the highest values of allergenic potency (VPA). These characteristics are presented by some of the most frequent species in Mediterranean urban environments such as *Acer negundo*, *Fraxinus excelsior*, *F. angustifolia*, *Populus alba* or *Platanus hispanica*. High allergenic value are presented by some of the species that are best adapted to Mediterranean climate conditions, such as different species of the Cupressaceae family (with *Cupressus* genus as the most prominent), Fagaceae family (sclerophyllous species of *Quercus*), and *Olea europaea*, that should be considered as ornamental species in addition to agronomic, due to its extensive presence in cities. Once the species-specific VPA was calculated, it was possible to apply the Urban Green Zones Allergenicity Index (I_UGZA_), which estimates the overall allergenic risk that these species represent in that the area where they grow. The 34 parks considered in this study are a good example of the different typologies that exist in Mediterranean cities—from urban forests to pocket parks and small plazas. The characteristics and factors that most affect the final allergenicity value were analyzed, highlighting the density of existing trees, the species richness and the Shannon index as the most significant factors. Environmental variables are also revealed as important parameters, since they affect the floristic composition of the parks, and this in turn affects the value of I_UGZA_. This information highlights the need to consider the allergenicity criterion as a parameter when managing, designing and planning current and future green areas, since only then can urban green areas be healthy spaces, inclusive for all population.

## Figures and Tables

**Table 1:** Results from the inventories of trees carried out in the Mediterranean parks considered in this study.

**SPECIES RICHNESS**	355 taxa (including species, sub-species, cultivars, hybrids)
**N° of FAMILIES**	83
**FAMILIES WITH THE LARGEST NUMBER OF GENERA**	
**Fabaceae**	14 (*Bauhinia*, *Ceratonia*, *Cladrastis*, *Cytisus*, *Erythrina*, *Gleditsia*, *Gymnocladus*, *Maackia*, *Parkinsonia*, *Robinia*, *Sophora*, *Tipuana*, *Vachellia*, *Wisteria*)
**Cupressaceae**	11 (*Calocedrus*, *Chamaecyparis*, *Cupressus*, *Cupressocyparis*, *Hesperocyparis*, *Juniperus*, *Metasequoia*, *Platycladus*, *Sequoiadendron*, *Tetraclinis*, *Thuja*)
**Rosaceae**	10 (*Cercocarpus*, *Cotoneaster*, *Crataegus*, *Cydonia*, *Eriobotria*, *Malus*, *Photinia*, *Prunus*, *Pyrus*, *Sorbus*)
**Fagaceae**	5 (*Castanea*, *Castanopsis*, *Fagus*, *Lithocarpus*, *Quercus*)
**Betulaceae**	5 (*Alnus*, *Betula*, *Carpinus*, *Corylus*, *Ostrya*)
**Pinaceae**	4 (*Abies*, *Cedrus*, *Pinus*, *Pseudotsuga*)
**Oleaceae**	4 (*Fraxinus*, *Ligustrum*, *Olea*, *Syringa*)
**GENERA WITH THE LARGEST NUMBER OF SPECIES**	
*Acer*	20 spp.
*Quercus*	16 spp.
*Betula*	12 spp.
*Pinus*	11 spp.
*Magnolia*	9 spp.
*Fraxinus*	8 spp.
*Populus*	8 spp.
*Prunus*	5 spp.
*Tilia*	5 spp.

**Table 2:** Reproductive characteristics, pollination, and permanence of leaf attributes in the tree species of the Mediterranean parks considered in this study.

**LOCATION OF REPRODUCTIVE ORGANS**	M.—On the same plant (Monoecious): 33.6% (119) D.—On different plants (Dioecious): 18.8% (67) H.—Having both sexes (Hermaphrodite): 28.6% (102) O.—Others (Polygamous, sub-dioecious, Parthenogenesis): 19.0% (67)
**POLLINATION STRATEGY**	I.—Insect-pollinated: 46.7% (166) W.—Wind-pollinated: 42.3% (150) A.—Ambiphillous: 11.0% (39)
**PERMANENCE OF THE LEAVES**	DE.—Deciduous: 65.0% (231) SD.—Semi-deciduous: 1.9% (7) EV.—Evergreen: 33.1% (117)

**Table 3:** Attributes, origin, allergenicity and hardiness zones of the 20 most-frequent species in Mediterranean parks.

Species	Attributes	Origin	Allergenicity Level	Hardiness Zone
*Acer campestre*	D-I-DE	Eur.As.Afr.	Moderate	6a–6b
*Acer pseudoplatanus*	H-I-DE	Eur.As.Afr.	Moderate	4b
*Acer negundo*	D-W-DE	N-Am.	High	4b
*Celtis australis*	M-W-DE	Eur (Med).	Moderate	5b
*Cupressus sempervirens*	M-W-EV	Eur (Med).	Very High	8b
*Fraxinus excelsior*	D-W-DE	Eur (Med).	High	4b–5a
*Fraxinus angustifolia*	D-W-DE	Eur (Med).	Moderate	6b–7b
*Ligustrum lucidum*	H-A-EV	China	Moderate	8a–11b
*Magnolia spp.*	H-I-EV	N-Am.China	Low	6b–8a
*Pinus halepensis*	M-W-EV	Eur (Med).	Low	8a–9b
*Pinus pinea*	M-W-EV	Eur (Med).	Low	6b
*Pinus pinaster*	M-W-EV	Eur (Med).	Low	8a
*Platanus x hispanica*	M-W-DE	Eur.	Very High	6b
*Populus alba*	D-W-DE	Eur.As.Afr.	High	4b
*Quercus ilex*	M-W-EV	Eur (Med).	Moderate	5b–7b
*Robinia pseudoacacia*	H-I-DE	N-Am.	Low	4b–5a
*Taxus baccata*	D-W-EV	Eur.	High	5a–5b
*Tilia cordata*	H-A-DE	Eur.	Low	5a
*Tilia platyphyllos*	H-A-DE	Eur. As.	Low	5a
*Ulmus minor*	D-W-DE	Eur. N-Am. As.	High	6b

Attributes: D: Dioecious; M: Monoecious; H: Hermaphrodite. I: Insect-pollinated; W: Wind-pollinated; A: Ambiphillous. DE: Deciduous; EV: Evergreen. Origin: Eur: Europe; As: Asia; Afr: Africa; N-Am: North-America; (Med): Mediterranean. China: China.

**Table 4:** Spearman rank order correlations between I_UGZA_ and environmental variables. Only variables with a significant correlation are shown (* *p* < 0.05, ** *p*< 0.01).

Variable	R
Tree density (ha.)	0.70 **
Number of trees	0.65 **
Number of species	0.54 **
Shannon index	0.45 **
Precipitation of May	0.43 *
Precipitation of July	0.38 *
BIO 18 (Precipitation of the warmest quarter)	0.35 *
Temperature of August	−0.34 *
BIO 1 (Annual mean temperature)	−0.35 *
BIO 9 (Mean temperature of the driest quarter)	−0.38 *

**Table 5:** Generalized Linear Model (GLM) results showing the most significant parameters correlated with the I_UGZA_ value.

Effect	Estimate	Standard	Wald	Lower CL	Upper CL	*p*
Intercept	0.541676	0.180138	9.0421	0.188612	0.894741	0.002638
Tree density(ha)	0.001736	0.000171	102.6181	0.001400	0.002072	0.000000
BIO1	−0.033637	0.011622	8.3761	−0.056416	−0.010857	0.003802

## Supplementary Materials

The following are available online at https://www.mdpi.com/1660-4601/16/8/1357/s1, Table S1: Name and type of park, locality, surface areas, number of species and trees, tree density, Shannon´s index and main contributors to I_UGZA_ of the green spaces included in this study.
