# Supplementary material for: Estimation of the Allergenic Potential of Urban Trees and Urban Parks: Towards the Healthy Design of Urban Green Spaces of the Future
Source: Int J Environ Res Public Health. 2019 Apr 15;16(8):1357. doi: 10.3390/ijerph16081357 (PMC6517926; doi:10.3390/ijerph16081357)
Supplement: Supplementary file 1 [file ijerph-16-01357-s001.pdf]

NAME OF PARK (TYPE)	LOCALITY (COUNTRY) COORDINATES	SURFACE AREA (m²)	SURFACE COVERED BY GRASS(m²)	Nº OF SPECIES	Nº OF TREES	DENSITY (TREES/HA.)	SHANNON´S INDEX	MAIN CONTRIBUTORS TO I_{UGZA}
Parco Stibbert (Community Park)	Firenze (Italy) 43º47´34´´N 11º15´17´´E	35 000	29 700	35	413	118	2,5	Cupressaceae, *Pinus spp., Quercus spp.*
Bosco dei Cento Pasii (Community Park)	Gaggiano, Milan (Italy) 45º25´12´´N 9º01´33´´E	100 000	45 000	15	7.710	771	3,2	*Carpinus betulus, Corylus spp., Fraxinus spp., Populus spp.*
Parco di Arlecchino (District Park)	Mantova (Italy) 45º11´14´´ N 10º53´54´´W	145 000	90 000	58	8.157	562,55	3,0	*Acer spp., Alnus spp., Carpinus betulus, Corylus spp., Populus spp., Quercus spp.*
Villa Revedin Bolasco (Historical Garden)	Castelfranco-Veneto (Italy) 45º40´26´´N 11º56´03´´E	80 000	40 000	71	1.516	189,5	3,1	*Carpinus betulus, Ligustrum spp., Taxus spp., Ulmus spp.*
Parco Centrale del Lago (District Park)	Rome (Italy 41º49´N 12º27¨W	165 130	68 176	50	935	56,63	3,3	Cupressaceae, *Laurus nobilis, Ligustrum spp., Populus spp., Taxus spp.*
Villa Chigi (Historic Urban)	Rome (Italy) 41º56´00´´N 12º30´56´´E	51 235	49 321	32	487	95,05	2,3	*Ailanthus altissima, Laurus nobilis, Olea europaea, Quercus spp.*
Parco Pincio (Historic Urban Promenade and Park)	Rome (Italy) 41º54´42´´N 12º28´41´´E	80 000	10 000	15	351	43,87	1,7	Cupressaceae, *Platanus x hispanica, Quercus ilex*
Villa Carpegna (Historic Urban Park)	Rome (Italy) 41º53´45´´N 12º25´36´´E	70 000	30 000	13	356	50,85	1,4	*Quercus ilex, Pinus spp.*

Parco Talenti (Contemporary Urban Park)	Rome (Italy) 41º57´8´´ N 12º33´24´´E	111 634	110 000	19	1.126	100,86	2,5	*Ailanthus altissima, Ligustrum spp., Olea europaea, Platanus x hispanica, Populus spp.*
Villa Lazzaroni (Historic Urban Park)	Rome (Italy) 41°52'20.6"N 12°31'25.4"E	63 625	40 000	54	397	62,39	3,1	Cupressaceae, *Laurus nobilis, Ligustrum spp., Pinus spp.*
Parco del Colle Opio (Modern Urban)	Rome (Italy) 41º53´71´´N 12º29´47´´E	115 000	60 000	13	385	33,47	2,1	Cupressaceae, *Olea europea, Pinus spp.*
Parco San Sebastiano (Urban Modern)	Rome (Italy) 41º52´36´´N 12º29´49´´E	65 000	20 000	13	288	44,30	2,0	*Carpinus betulus,* Cupressaceae, *Quercus spp., Ulmus spp.*
Villa Celimontana (Historic Urban Park)	Rome (Italy) 41º53´65´´N 12º29´43´´E	110 000	60 000	12	396	36	1,9	Cupressaceae, *Laurus nobilis, Pinus spp., Quercus ilex.*
Parco delle Mimose (Contemporary Urban Park)	Rome (Italy) 41º56´44´´N 12º32´3´´E	62 340	60 000	19	179	28,73	2,6	*Cupressus spp., Ligustrum spp., Olea europea, Pinus spp.*
Villa Sciarra (Urban Park)	Rome (Italy) 41º53´6´´N 12º27´53´´E	70 000	35 000	25	226	93,14	2,3	*Ailanthus altissima,* Cupressaceae, *Laurus nobilis,Quercus spp.*
Villa Paganini (Historical Park)	Rome (Italy 41º54´52´´N 12º30´38´´E	30 000	26 000	25	226	72,78	3,1	*Aesculus spp., Acer negundo, Fagus sylvatica.*
Jardin des Plantes (Historical)	Nantes (France) 47º13´09´´N 1º32´33.25´´W	70 000	40 000	160	420	60	4,1	*Alnus spp., Betula spp., Carpinus spp., Corylus spp., Quercus spp.*
Jardim Guerra Junqueiro/Jardin da Estela	Lisbon (Portugal) 38º42´53.2´´N -9º09´32´´W	46 915	8 250	119	645	88,04	4,1	*Ligustrum spp., Fraxinus spp., Olea europaea.*

Community Park)								
Parque da Paz (Modern District Park)	Almada (Setúbal, Portugal)<br>38º40´12´´N<br>9º09´29´´W	440 000	273 772	62	5,300	120,45	2,91	*Acer negundo*, Cupressaceae, *Fraxinus spp.*, *Ligustrum spp.*, *Olea europea*, *Platanus x hispanica*, *Quercus spp.*
Parque da Castillo (Historical Community Park)	Bragança (Portugal)<br>41.80466<br>-6.74962	37 200	20 000	35	404	108,6	2,9	*Betula spp.*, Cupressaceae, *Fraxinus spp.*, *Morus spp.*, *Ulmus spp.*
ISESCO (ex Murdoch) (Community Park)	Casablanca (Morocco)<br>33º34´25´´N<br>7º37´56´´ W	40 000	27 639	35	690	172,5	3,1	*Callistemum citrinus*, *Casuarina sp.*, Cupressaceae
Rambla de Belén (Boulevard)	Almería (Spain)<br>36º51´N<br>2º27´W	29 796	0	8	622	214,48	2,3	*Lagunaria patersonii*, *Ulmus minor*
Parque San Amaro (Historical Park)	Ceuta (Spain)<br>35º53´N<br>5º17´W	12 128	1 980	56	305	254,16	5,0	*Fraxinus spp.*, *Ginkgo biloba*, *Olea europaea*
Jardín de la Agricultura (Historical)	Córdoba (Spain)<br>37º53´N<br>4º47´W	30 542	6 108	32	356	118,66	4,5	*Platanus hispánica*, *Phoenix spp.* *Citrus sinensis*
Parque García Lorca (Urban Park)	Granada (Spain)<br>37º10´N<br>3º34´W	71 500	24 000	77	788	110,98	4,4	*Cupressaceae*, *Ginkgo biloba*, *Morus alba*, *Olea europaea*
Parque Miguel Servet (Urban)	Huesca (Spain)<br>42º08´N<br>0º24´W	65 500	4 545	62	1725	265,38	4,0	*Carpinus betulus*, Cupressaceae, *Ligustrum spp.* *Patanus x hispanica,Tamarix spp.*

Parque Santa Margarita (Community Park)	La Coruña (Spain) 43º21´56´´N 8º24´41´´ W	87 500	37 246	59	1056	120,68	2,3	*Acer spp., Fagus sylvatica, Ligustrum spp., PLatanus x hisanica, Quercus spp., Ulmus spp.*
Las Alamedas (Urban Park)	Lorca (Spain) 37º39´N 01º41´W	25 800	1 000	32	597	231,39	3,5	*Platanus x hispanica, Ulmus sp., Acer negundo, Cupressus macrocarpa, Populus spp.*
Parque El Retiro (Historical Park)	Madrid (Spain) 40º24´N 3º41´W	1 180 000	373 941	147	19.022	161,20	3,9	*Aesculus hippocastanum, Broussonetia papyrifera, Carpinus betulus,* Cupressaceae, *Platanus hispanica*
Parque San Francisco (Historical Park)	Oviedo (Spain) 43º21´N 5º21´W	90 000	12 000	57	836	92,88	4,0	*Aesculus hippocastanum, Fraxinus spp., Platanus hispanica, taxus spp.*
Parque La Taconera (Historical Park)	Pamplona (Spain) 42º49´N 1º39´W	90 000	60 000	53	1333	148,11	4,2	*Aesculus hippocastanum, Betula spp., Fraxinus spp., Platanus x hispanica.*
Parque Las Llamas (Urban Park)	Santander (Spain) 43º28´N 3º48´W	11 000	40 000	57	1709	155,36	5,3	*Alnus spp., Betula spp., Quercus spp.*
Jardín de Ayora (Historical Garden)	Valencia (Spain) 39º28´N 0º20´W	22 000	2 000	44	442	200	4,6	*Acer negundo, Ligustrum spp., Casuarina sp.,* Cupressaceae.
Miklošičev Park (Community Park, Historical)	Ljubljana (Slovenia) 46,054270°N 14,506546°W	4 374	3 622	9	38	86,87	1,7	*Pterocarya fraxinifolia, Tilia cordata.*

Table S1: Name and type of park, locality, surface areas, number of species and trees, tree density, Shannon´s index and main contributors to I_{UGZA} of the green spaces included in this study.
